# Newly emerging diseases of marine turtles, especially sea turtle egg fusariosis (SEFT), caused by species in the *Fusarium solani* complex (FSSC)

**DOI:** 10.1080/21501203.2019.1710303

**Published:** 2020-01-07

**Authors:** Frank H. Gleason, Monika Allerstorfer, Osu Lilje

**Affiliations:** School of Life and Environmental Sciences, University of Sydney, Sydney, Australia

**Keywords:** Fungal infections, plant parasites, sea turtle egg fusariosis, floating plastic substrates, global temperature rise, Neocosmospora

## Abstract

Sea turtles are presently considered severely endangered species that are historically threatened by many environmental factors. Recently, additional threats to sea turtles from two pathogenic species of fungi in the *Fusarium solani* species complex (*F. falciforme* and *F. keratoplasticum*) have been identified. These species infect marine turtle eggs, causing sea turtle egg fusariosis, and kill their embryos, with recent reports of hatch-failure in seven globally distributed species of endangered sea turtles (*Caretta caretta, Chelonia mydas*, Dermochelys *coriaceae, Eretmochelys imbricata, Lepidochelys olivacea, Lepidochelys kempi* and *Natator depressus*). Mycelia and spores of pathogenic species of *Fusarium* are produced in disturbed terrestrial soils and are transported to the ocean in coastal run off. We propose that these fungi grow on floating particles of plant tissues (leaves and wood), animal tissues, silt and plastics, which are carried by wind and currents and the turtles themselves to the beaches where the turtles lay their eggs.

## Introduction

In marine ecosystems the prevalence of infectious diseases caused by fungi has dramatically increased during the past two decades, likely due to the transmission of emerging pathogens into new environments and the rapid rate of global climate change (Altizer et al. 2003; Harvell et al. 2009; Groner et al. 2016). In fact, emerging infectious diseases are at present one of the main threats to global biodiversity (Altizer et al. 2003; Bax et al. 2003; Harvell et al. 2009; Hawkes et al. 2009; Lafferty 2009). Sea turtles are a well-known example of an endangered species whose populations are presently being significantly reduced in size by fungal infections (Sarmiento-Ramirez et al. 2014a; Reynolds et al. 2017). The general topics of turtle health and the effects of climate change on turtle populations were recently reviewed by Flint (2013) and by Hamann et al. (2013). The present review includes new information made available since these two reviews were published.

A large number of species of fungi infect both marine and freshwater species of juvenile and adult turtles and their eggs causing infectious diseases (Tables 1 and 2). In Table 1 we summarised reported fungal pathogens found recently in sea turtle species, to give an overview of how widely distributed fungal pathogens are within these marine animals and which species have been infected so far. The most abundant fungal orders are the Eurotiales and Hypocreales. The *Fusarium* species found in sea turtles and their nests belong to the order Hypocreales.

**Table 1.** Identified *Fusarium* and other fungal pathogens of sea turtles.

Host species	Fungal species	Host origin and date of first observation	Place of detection	References
Family: Cheloniidae				
loggerhead sea turtle (*Caretta caretta*)	*Fusarium solani*	1996, Barcelona, Spain	found in the wild offshore, brought into captivity for rehabilitation	Cabanes et al. 1997
loggerhead sea turtle (*Caretta caretta*)	*Fusarium solani*	Bimini, Bahamas, USA	captivity	Cabanes et al. 1997
loggerhead sea turtle (*Caretta caretta*)	*Fusarium solani*	Boavista, Cap Verde, Africa	wild	Sarmiento-Ramirez et al. 2010
loggerhead sea turtle (*Caretta caretta*)	*Paecilomyces sp., Penicillium sp., Aspergillus sp., Fusarium sp.*	turtles obtained from farms on Badu, Sue, Yam, Coconut and Yorke islands in the Torres Strait and from an oceanarium on Magnetic Island near Townsville, Australia	captivity	Glazebrook et al. 1993
loggerhead sea turtle (*Caretta caretta*)	*Fusarium solani, Pseudallescheria boydii*	Mon Repos Conservation Park, Bundaberg; 1997-1998	wild	Phillott and Parmenter 2001
loggerhead sea turtle (*Caretta caretta*)	*Aspergillus* sp., *Chrysosporium* sp., *Fusarium* sp. and *Penicillum* sp. were identified in nests and eggshells; Absidia sp., Calindrocarpon sp., Emericella sp. and Mucor sp. were only identified in nests; Cladosporium sp. and Thielavia sp. were only identified in eggshells;	Fethiye beach, Turkey; 2004	wild	Gücül et al. 2010
loggerhead sea turtle (*Caretta caretta*)	*Fusarium falciforme, Fusarium keratoplasticum*	Jekyll Island, GA, USA; 2010 and 2012;	wild	Brofft Bailey et al. 2018
loggerhead sea turtle (*Caretta caretta*)	*Fusarium oxysporum, Fusarium solani, Pseudallescheria boydii*	Heron Island, Wreck Island, Peak Island, Milman Island, Mon Repos Conservation Park; 1996/1997 & 1998/1999	wild	Phillott et al. 2004
green sea turtle (*Chelonia mydas*)	*Paecilomyces* sp., *Penicillium* sp., *Aspergillus* sp., *Fusarium* sp.	turtles obtained from farms on Badu, Sue, Yam, Coconut and Yorke islands in the Torres Strait and from an oceanarium on Magnetic Island near Townsville, Australia	captivity	Glazebrook et al. 1993
green sea turtle (*Chelonia mydas*)	*Fusarium* sp., *Fusarium solani* species complex (FSSC), *Fusarium oxysporum, Fusarium proliferatum*	Terengganu and Melaka, Malaysia; 2010	wild	Sidique et al. 2017
green sea turtle (*Chelonia mydas*)	*Fusarium oxysporum, Fusarium solani, Pseudallescheria boydii*	Heron Island, Australia;	wild	Phillott and Parmenter 2006
green sea turtle (*Chelonia mydas*)	*Fusarium solani, Pseudallescheria boydii*	Heron Island, Australia; 1996-1997	wild	Phillott and Parmenter 2014
green sea turtle (*Chelonia mydas*)	*Fusarium solani, Pseudallescheria boydii*	Heron Island, Australia; 1997-1998	wild	Phillott and Parmenter 2001
green sea turtle (*Chelonia mydas*)	*Fusarium falciforme, Fusarium keratoplasticum, Pseudallescheria* sp., *Scedosporium* sp., *Aspergillus* sp., *Phoma* sp., *Alternaria* sp., *Gymnascella* sp., *Pleosporales* sp.	English bay, North East bay, Pan Am beach and Long beach at Ascension Island;	wild	Sarmiento-Ramirez et al. 2017
green sea turtle (*Chelonia mydas*)	*Fusarium oxysporum, Fusarium solani, Pseudallescheria boydii*	Heron Island, Wreck Island, Peak Island, Milman Island, Mon Repos Conservation Park; 1996/1997 & 1998/1999	wild	Phillott et al. 2004
green sea turtle (*Chelonia mydas*)	*Paecilomyces* sp., *Fusarium scirpi, Penicillium* sp.	turtle farms in the Torres Strait; April 1977 to September 1980; two hawksbill turtles from a group of experimental specimens on Yorke Island	captivity	Glazebrook & Campbell 1990
green sea turtle (*Chelonia mydas*)	*Aspergillus flavus, Aspergillus fumigatus, Asperigllus nidulans, Aspergillus niger, Aspergillus terreus, Aspergillus ochraceus, Cladosporium cladosporoides, Emericella nidulans, Eurotium amstelodami, Eurotium rubrun, Fusarium moniliforme, Penicillium* sp., *Rhizopus stolonifer, Trichoderma viride*	Ras Al Jinz Reserve, Oman,Asien; 2003	wild	Elshafie et al. 2007
hawksbill turtle (*Eretmochelys imbricata*)	*Fusarium* sp., *Fusarium solani* species complex (FSSC), *Fusarium oxysporum, Fusarium proliferatum*	Terengganu and Melaka, Malaysia; 2010	wild	Sidique et al. 2017
hawksbill turtle (*Eretmochelys imbricata*)	*Fusarium oxysporum, Fusarium solani, Pseudallescheria boydii*	Milman Island, Australia;	wild	Phillott and Parmenter 2006
hawksbill turtle (*Eretmochelys imbricata*)	*Fusarium falciforme*	La Playita beach at Machalilla National Park, Ecuador; 2012	wild	Sarmiento-Ramirez et al. 2014b
hawksbill turtle (*Eretmochelys imbricata*)	*Aspergillus terreus, Aspergillus niger, Aspergillus flavus, Cladosproium cladosporioides, Nigrospora grisea, Fusarium solani, Fusarium lateritium, Fusarium oxysporum*	beaches of Muro Alto, Cupe and Merepe, Pernambuco State, Brazil;	wild	Neves et al. 2015
hawksbill turtle (*Eretmochelys imbricata*)	*Fusarium oxysporum, Fusarium solani, Pseudallescheria boydii*	Heron Island, Wreck Island, Peak Island, Milman Island, Mon Repos Conservation Park; 1996/1997 & 1998/1999	wild	Phillot et al. 2004
hawksbill turtle (*Eretmochelys imbricata*)	*Paecilomyces* sp., *Fusarium scirpi, Penicillium* sp.	turtle farms in the Torres Strait; April 1977 to September 1980; two hawksbill turtles from a group of experimental specimens on Yorke Island	captivity	Glazebrook & Campbell 1990
olive ridley turtle (*Lepidochelys olivacea*)	*Saksenaea* sp., *Aspergillus* sp., *Fusarium* sp., *Cladosproium* sp., *Mucor* sp., *Allescheria* sp., *Acremonium* sp., *Penicillium* sp.	Nancite beach, Costa Rica; 1987 – 1991	wild	Mo et al. 1992

**Table 2.** Species of fungi other than *Fusarium* spp. infecting sea turtles.

Fungal species	Turtle species
*Sporotrichium sp.*	*Chelonia mydas*
*Cochliobolus sp.*	*Chelonia mydas*
*Cladosporium sp.*	*Caretta caretta, Chelonia mydas, Lepidochelys olivacea, Dermatochelys coriacea*
*Cladosporium cladosporoides*	*Caretta caretta, Eretmochelys imbricata, Chelonia mydas*
*Alternaria sp.*	*Chelonia mydas*
*Alternaria arborescens*	*Caretta caretta*
*Ampelomyces sp.*	*Caretta caretta*
*Phoma sp.*	*Chelonia mydas*
*Ochroconis sp.*	*Chelonia mydas*
*Eurotium amstelodami*	*Chelonia mydas*
*Eurotium rubrum*	*Chelonia mydas*
*Aspergillus sp.*	*Caretta caretta, Chelonia mydas, Lepidochelys olivacea, Dermatochelys coriacea*
*Aspergillus flavus*	*Eretmochelys imbricata, Chelonia mydas*
*Aspergillus fumigatus*	*Chelonia mydas*
*Aspergillus nidulans*	*Chelonia mydas*
*Aspergillus niger*	*Eretmochelys imbricata, Chelonia mydas*
*Aspergillus terreus*	*Eretmochelys imbricata, Chelonia mydas*
*Aspergillus ochraceus*	*Chelonia mydas*
*Emericella sp.*	*Caretta caretta, Chelonia mydas*
*Emericella nidulans*	*Chelonia mydas*
*Paecilomyces sp.*	*Caretta caretta, Chelonia mydas, Eretmochelys imbricata, Lepidochelys olivacea*
*Paecilomyces fumosa-roseus*	*Chelonia mydas*
*Penicillium sp.*	*Caretta caretta, Chelonia mydas, Eretmochelys imbricata, Lepidochelys olivacea, Dermatochelys coriacea*
*Chrysosporium sp.*	*Caretta caretta*
*Gymnascella sp.*	*Chelonia mydas*
*Allescheria sp.*	*Lepidochelys olivacea, Dermatochelys coriacea*
*Oospora sp.*	*Chelonia mydas*
*Absidia sp.*	*Caretta caretta*
*Actinomucor sp.*	*Chelonia mydas*
*Mucor sp.*	*Caretta caretta, Lepidochelys olivacea, Dermatochelys coriacea*
*Rhizopus sp.*	*Chelonia mydas*
*Rhizopus stolonifer*	*Chelonia mydas*
*Apophysomycetes sp.*	*Chelonia mydas*
*Saksenaea sp.*	*Lepidochelys olivacea, Dermatochelys coriacea*
*Candida albicans*	*Caretta caretta*
*Colletotrichum acutatum*	*Lepidochelys kempi*
*Beauveria bassiana*	*Caretta caretta*
*Acremonium sp.*	*Lepidochelys olivacea, Dermatochelys coriacea*
*Trichoderma viride*	*Chelonia mydas*
*Cylindrocarpon sp.*	*Caretta caretta*
*Purpureocillium lilacinum*	*Caretta caretta, Chelonia mydas*
*Pseudallescheria sp.*	*Chelonia mydas*
*Pseudallescheria boydii*	*Caretta caretta, Chelonia mydas, Eretmochelys imbricata, Natator depressus*
*Scedosporium sp.*	*Chelonia mydas*
*Thielavia sp.*	*Caretta caretta*
*Nigrospora grisea*	*Eretmochelys imbricata*
*Pleosporales sp.*	*Chelonia mydas*
*Veronaea botryosa*	*Chelonia mydas*

The primary focus of the present review is the ecology of pathogenic fungi in the *Fusarium solani* species complex (FSSC) and the relationship of these fungi to disease in marine turtles. A newly emerging fungal disease, sea turtle egg fusariosis (STEF), linked to sea turtle egg mortality in sea turtle nests worldwide, is caused by members of one lineage, the *F. solani* species complex (FSSC). The two common and cosmopolitan *Fusarium* species *F. keratoplasticum* and *F. falicforme* are known to be associated with STEF (Smyth et al. 2019). *Fusarium solani* Mart (Sacc) is defined on the basis of morphological characteristics but molecular investigations show that FSSC is actually a diverse complex of many phylogenetically distinct species (Short et al. 2013). The turtle-infecting strains of *Fusarium* have been placed by Zhang et al. (2006a) in the *F. solani* species complex group 2, which consists of many human, animal, and plant pathogens as well as environmental isolates (Sandoval-Denis et al. 2019).

*Fusarium keratoplasticum* Geiser, O’Donnell, Short *et* Zhang and *F. falciforme* (Carrion) Summerb. et Schroers cause a potentially lethal disease in many species of marine turtles (Sarmiento-Ramirez et al. 2014a, 2017). These two species of fungi infect the eggs of marine turtles in their nests resulting in low hatching success and subsequently lower population sizes in the oceans. *F. falciforme* and *F. keratoplasticum* have been implicated in hatch-failure in the seven globally distributed species of sea turtles (*Caretta caretta, Chelonia mydas, Dermochelys coriaceae, Eretmochelys imbricata, Lepidochelys olivacea, Lepidochelys kempi* and *Natator depressus*) (Sarmiento-Ramirez et al. 2014a).

The FSSC is thought to have a world-wide distribution (Sarmiento-Ramirez et al. 2017; Sandoval-Denis et al. 2019) and appears to include some of the world’s most destructive pathogens, infecting both economically and ecologically important plant and animal hosts. These pathogens are found growing actively in multiple environments, including sink drains, human tissues, soil, silt, contact lenses and as saprotrophs in rich organic agricultural soils and in many other aquatic and terrestrial environments (environmental isolates) (Zhang et al. 2006b and 2006c; Short et al. 2013; O’Donnell et al. 2015). A number of *Fusarium* species have been detected on sea turtles and their eggs. Eight different *Fusarium* species have been found associated with sea turtles and their nesting beaches (Table 2).

**Figure 1.**
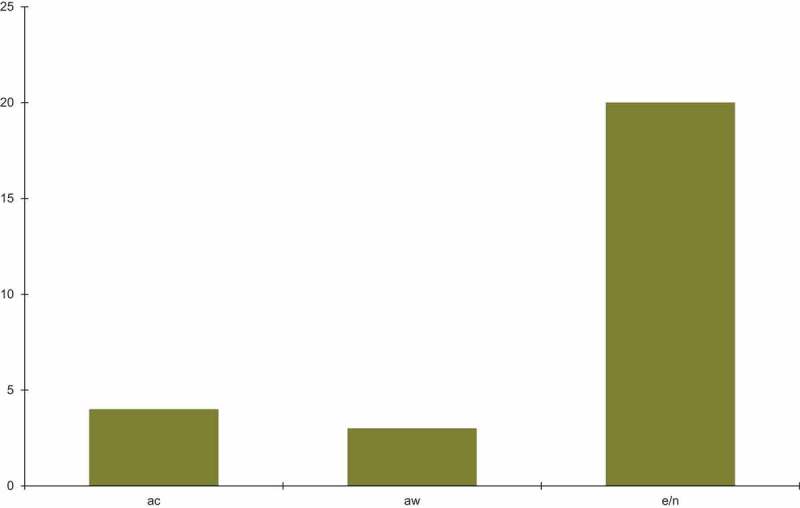
The total number of cases of *Fusarium* infection in captive and wild sea turtles and their eggs/nests. The data are taken from Table 1. The following abbreviations are used: ac-animals in captivity, aw-animals in the wild, e/n-eggs/nests.

**Figure 2.**
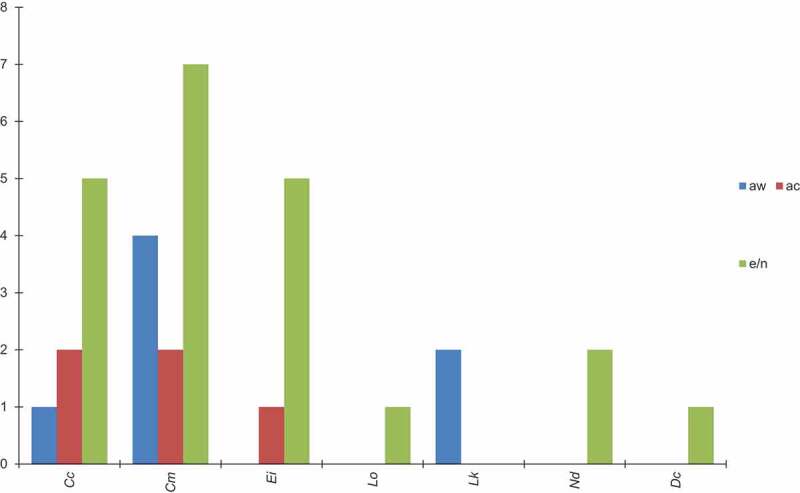
The numbers of cases of *Fusarium* infections in wild and captive species and their nests/eggs. The data are taken from Table 1. The following abbreviations are used: Cc-*Caretta caretta*, Cm-*Chelonia mydas*, Ei-*Eretmochelys imbricata*, Lo-*Lepidochelys olivacea*, Lk-*Lepidochelys kempi*, Nd-*Natator depressus*, Dc-*Dermatochelys coriacea*, aw-animals in the wild, ac-animals in captivity, e/n-eggs/nests.

**Figure 3.**
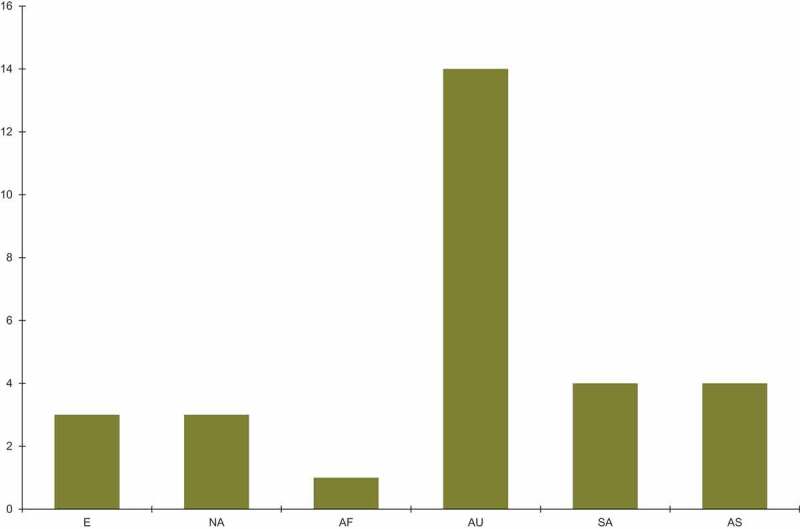
A comparison of *Fusarium* infections only in sea turtle species found all over the world. The data are taken from Table 1. The following abbreviations are used: E-Europe, NA-North America, AF-Africa, AU-Austrialia, SA-South America, AS-Asia.

*Fusarium* is the major source of infection of sea turtle eggs and nests. Infections by *Fusarium* species (Figure 1) have been reported in nests and eggs of sea turtle for both wild and captive sea turtles. Recently reported *Fusarium* infections according to sea turtle species are summarised in Figure 2. The majority of reports come from loggerhead, green and hawksbill turtles and were from Australia (Figure 3). Other parts of the world including Europe, North and South America and Asia have 3–4 reports, followed by Africa with only one reported case.

Plant diseases caused by species of *Fusarium* pose a significant threat to the human food supply and agricultural biosecurity in soil and in other terrestrial ecosystems (O’Donnell et al. 2015). Species of *Fusarium* can be a threat to marine animals in aquaculture as well, such as in commercial culture of the lobster (*Homarus vulgaris*) (Alderman 1981). The scope of the present review is restricted to diseases of marine turtle eggs caused by fungi in the FSSC.

## Classification

The genus *Fusarium* contains a very large number of species, one of which is the species *F. solani*. The classification of the species *Fusarium solani* is complicated as well, and probably this group should be divided into many species, subspecies or varieties based on differences in their genotypes. The fungi in the FSSC are assigned to the Family Nectriaceeae (Order Hypocreales, Class Sordariomycetes, Subphylum Eurotiomycetes, and Phylum Ascomycota), based on phylogenetic studies (Spatafora et al. 2006; Zhang et al. 2006a). O’Donnell et al. (2009) reviewed the status of the classification of all of the described species in the genus *Fusarium* which are pathogenic to turtles.

Sarmiento-Ramirez et al. (2017) subdivided all of their isolates of species of *Fusarium* collected from eggshells of turtles (*Chelonia mydas*) from sites near Ascension Island into three clades and three subclades in an out-group rooted cladogram of the ITS mDNA regions of their isolates. All of the *F. keratoplasticum* isolates were placed into clade III, subclade C, and all of the isolates of F. *falciforme* were placed into clade III, subclade A.

In another study, Sidique et al. (2017) constructed a phylogenetic tree of species of *Fusarium* pathogenic to turtles. The fungi were isolated from eggshells of two species of turtles (*Chelonia mydas* and *Eretmochelys imbricata*), nest sands, debris and roots near nest sites in Malaysia. The patterns of clades were different from the study by Sarmiento-Ramirez et al. (2017). Based on phylogenetic analysis of nucleotide sequences of the translation elongation factor 1-alpha gene, the FSSC isolates were separated into two main clades I and II with Main Clade I consisting of four sub-clades (A-D) and Main Clade II consisting of two sub-clades (E and F) (Sidique et al. 2017).

The presence of multiple clades of *Fusarium solani* in the sea turtle populations indicates genetic diversity within this group of fungal pathogens.

Sandoval-Denis et al. (2019) reviewed the very recent changes to the classification of *Fusarium*. Species of *Fusarium solani* which are pathogens of sea turtles presently have been reassigned to the genus *Neocosmospora*. For example, *Neocosmospora keratoplastica* which was isolated from sea turtles is an opportunistic pathogen (Sandoval-Denis et al. 2019).

## The aetiology of sea turtle egg fusariosis

Pathogenic fungi grow in the nests of marine turtles by initially forming mycelial networks on damaged eggs. These mycelial networks subsequently cover the surface of the eggs, produce enzymes and organic acids which degrade the shells by dissolving the organic substrates and calcium carbonate and finally penetrate the viable tissues inside. Infected eggs develop yellowish-blue infection zones that eventually become necrotic lesions and eventually kill the living embryos itself (Phillott et al. 2006; Sarmiento-Ramirez et al. 2010).

The eggs appear to become infected through contact with the sand around the nests and by gravid turtles and are less susceptible in dry sand than sand in intertidal zones or clay/silt soils (Abella-Pérez 2011; Phillott et al. 2002; Sarmiento-Ramirez et al. 2014b). Infected eggs within a clutch can serve as a source of inoculum for neighbouring eggs, with infection beginning on non-viable eggs (Phillott and Parmenter 2001).

Because of the presence of microorganisms in the egg chamber sand, it has been suggested that sea turtle cloacal fluid contains antimicrobial properties to protect the developing eggs (Keene et al. 2014). This clear, sometimes viscous, fluid contains glycoproteins and coats eggs as it is secreted from the cloaca during egg deposition into the nest chamber. As such, olive ridley turtles deposit 250–500 ml of fluid in a single nest, which could potentially provide protection if antimicrobials are present in the fluid (Keene et al. 2014).

The warm, moist environment and presence of organic matter at the nesting site of sea turtles is ideal for the growth of soil fungi that contribute to the hatching failure of the eggs either by decomposing the eggs and/or secreting mycotoxins that negatively affect the developing embryos. Fungi implicated in egg failure have been isolated from soil at nesting sites; exterior and/or interior of unhatched eggs; and embryonic tissue of the eggs. Although sandy soil from the nesting sites of the sea turtles often lack an adequate amount of organic matter for the rapid growth of fungi. Large amounts of organic matter represented by egg shells of hatched and failed eggs are added annually to the nesting sites (Bézy et al. 2014). This creates an optimum medium for fungal growth and sporulation that subsequently contributes to egg contamination. (Abella-Pérez 2011; Phillott and Parmenter 2001; Sarmiento-Ramirez et al. 2014b).

The most common fungi reported from soil at the nesting sites and from failed eggs were species in the genera *Aspergillus* and *Fusarium* (*Neocosmospora*). These fungi were found growing on 29% and 4% of the failed and hatched eggs respectively. Fungal hyphae were found growing on the surface of failed eggs, inside the eggshells, and on the egg membranes (Elshafie et al. 2007). Although some isolates are known to be potential pathogens, the presence of these fungi does not necessarily lead to the development of disease. During embryonic, development, the eggs are incubated for a long period covered by sand under conditions of high humidity and constant warm temperature, which are known to favour the growth of soil-borne fungi such as *F. solani*. However, these conditions may not be the only factors determining disease development (Sarmiento-Ramirez et al. 2010). Sarmiento-Ramirez et al. (2010) have also examined and detected the presence of *F. solani* in nests with asymptomatic eggs. This suggests that other factors such as specific microclimatic conditions, sand composition and natural immunosuppression influence the development of disease. The developing immune system gains full maturity and competence only during and after embryonic development of embryos. In addition, immunosuppression, e.g. due to accumulation of toxic substances in turtles and their eggs, etc. may be determining the development of the disease.

The close proximity of eggs within the sea turtle nest could also allow fungal growth to influence eggs without direct contact. For example, volatile mycotoxins or other metabolites, that originate from fungal growth on the exterior of one egg, could affect adjacent eggs, and could have a detrimental effect on the development and condition of hatchlings (Phillott and Parmenter 2014).

Marine turtles, while feeding in the ocean from the surface down to and including the benthos, frequently ingest a variety of solid particles including insoluble inorganic particles (such as silt), insoluble organic particles (such as plant leaves and wood and tissues of animal origin) and microplastic particles (Duncan et al. 2018; Matiddi et al. 2017). Species of *Fusarium* and *Aspergillus* which are pathogenic to animals including humans are known to colonise these substrates (Das and Kumar 2014; Oberbeckmann et al. 2015). If *F. solani* spores or hyphae are attached to these particles, viable fungal cells can enter the digestive system during feeding, go through the digestive system and be left in faeces in the turtle nests where they can grow on fried cellulose containing plant substrates (Keene et al. 2014).

## The possible roles of mycotoxins and proteases excreted by *Fusarium* and protease inhibitors excreted by turtles in diseases of marine turtle eggs

Species of fungi which are plant pathogens or which digest plant cell wall materials saprotrophically in the soil would be expected to excrete enzymes which demonstrate high effectiveness for natural lignocellulosic biomass degradation and utilisation. In particular, species of *Fusarium* are indeed robust cellulose and hemicellulose degraders and excrete extracellular cellulases and xylanases which are stable over the wide pH and temperature ranges normally found in the soil (Huang et al. 2015). These pathogens have optimal growth temperatures of 28–29.7°C, similar to the temperature requirements of sea turtle embryos. Successful incubation of sea turtle eggs requires a narrow thermal range of 25°C to 35°C with varying tolerances between species and populations of sea turtles (Mrosovsky et al. 1992; Montero et al. 2019).

In addition, many species of Ascomycota, including some species of *Fusarium*, are known to excrete mycotoxins. Mycotoxins can be toxic to many species of animals which are either directly infected by pathogenic species of *Fusarium* (or by other Ascomycota) or which eat food resources such as plant materials infected by one of the mycotoxin-producing species. This topic has recently been reviewed by Duan et al. (2016), Azliza et al. (2014) and Lemmens (2012).

It is possible that mycotoxins excreted by *Fusarium solani* and other fungi growing on the surface of egg shells might diffuse into tissues of the embryos ultimately causing death. In fact, some species of *Fusarium* are known to produce a number of mycotoxins in culture, including fumonisins. If these toxins are produced in and on the surface of eggs they can affect embryo development (Elshafie et al. 2007). Elshafie et al. (2007) found aflatoxins in 40% of the eggshells studied at a concentration ranging between 4.1 and 8.4 ppb and in 25% of failed egg’s contents at concentrations of 0.14–2.0 ppb. This level of aflatoxins in eggshells and egg contents is high enough to cause embryo mortality (Elshafie et al. 2007).

Proteases excreted by *F. solani* and other fungi might digest embryo cells as well. Olivieri et al. (2002) characterise a serine protease excreted by *F. solani*. Lowe et al. (2015) found that *F. graminearum* excretes a complex suite of extracellular proteases including metalloprotease, cysteine, serine, threonine and aspartate proteases.

However, some turtles have other defensive strategies to prevent disease. For example, they can protect their embryos from damage by proteases because their egg whites contain protease inhibitors. Ray et al. (1982) purified and characterised an acidic trypsin/subtilisin inhibitor in turtle eggs.

## Other species of fungi infecting sea turtles

**Figure 4.**
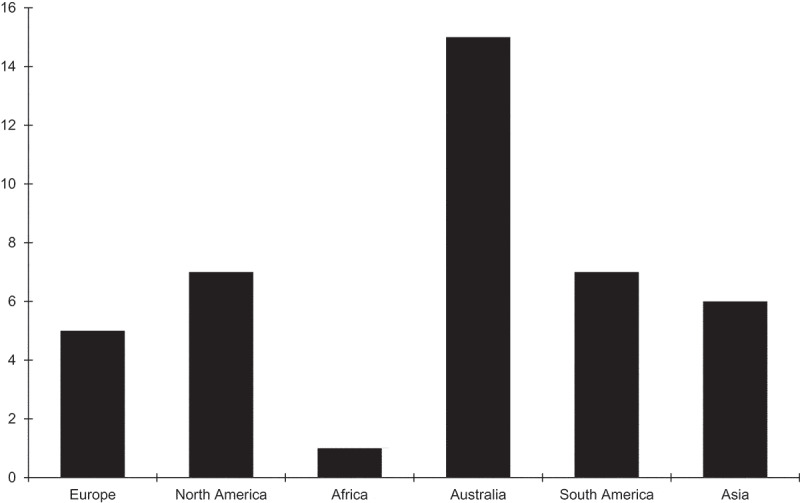
Sea turtle species infected with fungal pathogens including *Fusarium* found in the ocean near the continents. The data are taken from Table 1.

Species in the FSSC are not the only fungi found to penetrate sea turtle egg shells. For example, Candan (2018) also identified several species of *Aspergillus, Emericella, Rhizopus, Actinomucor*, and *Apohysomyces* in the nests of living green turtles using molecular methods. There are reports of fungal pathogens of sea turtles in many parts of the world, including Australia, North America, South America, Asia, Europe and Africa (Table 1). In Figure 4, we show the distribution of diseases caused by fungal pathogens of turtle species in the world. Fungal infections of the loggerhead (*Caretta caretta*) have been reported around the world. The reports of fungal infections in the green turtle (*Chelonia mydas*) have not been found in Africa and South America. No reports of the hawksbill turtle (*Eretmochelys imbricata*) are known from Europe, North America and Africa. Fungal infections of olive ridley (*Lepidochelys olivacea*) and the leatherback turtle (*Dermatochelys coricea*) have only been reported in South America, whereas reports of Kemp’s ridley (*Lepidochelys kempi*) have come from North America and of the flatback turtle (*Natator depressus*) only from Australia.

Species of fungi which appear to be pathogenic to turtle eggs have been observed in the fossil record. For example, Jackson et al. (2009) observed a species of *Penicillium* penetrating the shells of fossilised eggs from a lower Cretaceous geological formation in China. Fungal skin diseases are thought to be a major concern to the health of captive marine turtles (Orós et al. 2004b). All fungi so far identified from turtle skin lesions are common saprophytes (Wiles and Rand 1987). *Fusarium solani, Aspergillus* sp., *Geotrichum* sp., *Penicillium* sp., *Scolecobasidium* sp., *Fusarium* sp., *Drechslera* sp. and several unidentified fungi have been isolated from skin lesions in sea turtles.

Turtles can also be infected by systemic diseases such as mycotic granulomas (Orós et al. 2004a). Although mycotic granulomas can be found in the liver and throughout the coelomic cavity of wild and captive sea turtles, systemic mycotic infections occur primarily in the lungs. In captivity water quality appears to be an important factor in the incidence of cutaneous diseases due to the crowded conditions in the husbandry tanks (Orós et al. 2004a). The increased amount of nutrients in the water favour the growth of fungi in general.

## Discussion and conclusions

The spread of fungal diseases is a serious threat to biodiversity especially due to the increasing number of pathogenic fungi that threaten wildlife and domesticated species worldwide (Sidique et al. 2017). Most fungal infections in chelonias are considered opportunistic and secondary to underlying health conditions or accelerated by immunosuppression. Factors that may predispose an individual include suboptimal environmental temperatures and humidity, nutritional imbalances, infections by other pathogens, and trauma (Donnelly et al. 2015). But not only adult animals in the ocean are threatened by fungal pathogens, the nests of chelonians and the eggs are especially susceptible to fungi. According to some researchers, the hatching success depends upon the interaction of a number of factors such as salinity, humidity, temperature, gas flow, rainfall, tidal inundation, erosion and predation (Özdemir and Türkozan 2006). The recognition of the FSSC as a potential global threat to sea turtle eggs represents another instance of the importance of an emerging fungal infection impacting on wildlife (Bailey et al. 2018).

In the present review we propose mechanisms for the transport of conidia and hyphae of the pathogenic species of *Fusarium* produced in disturbed soils in terrestrial ecosystems to the turtle eggs at the beach. Pathogenic species of fungi can be transported from terrestrial ecosystems to the ocean primarily in run off. These fungi can grow on floating particles of plant tissues, silt and plastics in the ocean which are carried by wind and currents to the beaches where the turtles lay their eggs. These fungi are also carried by the turtles themselves in the digestive system (Clukey et al. 2018; Duncan et al. 2018).

## Highlights

- Seven species of sea turtles are presently considered severely endangered species
- *Fusarium falciforme* and *F. keratoplasticum* can infect turtle eggs killing the embryos
- Hatch-failure has recently been reported in the globally distributed species
- These fungi grow on floating particles of plant tissues, silt and plastics in the ocean

## References

[cit0001] Abella-Pérez E. 2011. Environmental and management factors affecting embryonic development in the loggerhead turtle *Caretta caretta* (L., 1758): implications for controlled egg incubation programmes. Zoologia Caboverdiana. 2(1):40–42.

[cit0002] Alderman DJ. 1981. *Fusarium solani* causing exoskeletal pathology in cultured lobsters, *Homarus vulgaris*. Trans Br Mycol Soc. 76:25–27.

[cit0003] Altizer S, Harvell D, Friedle E. 2003. Rapid evolutionary dynamics and disease treats to biodiversity. Trends Ecol Evol. 18:589–596.

[cit0004] Azliza IN, Hafizi R, Nurhazrati M, Salleh B. 2014. Production of major mycotoxins by *Fusarium* species isolated from wild grasses in peninsular Malaysia. Sains Malaysianna. 43:89–94.

[cit0005] Bailey JB, Lamb M, Walker M, Weed C, Craven KS. 2018. Detection of potential fungal pathogens *Fusarium falciforme* and *F. keratoplasticum* in unhatched loggerhead turtle eggs using a molecular approach. Endangered Species Res. 36:111–119.

[cit0006] Bax N, Williamson A, Aguero M, Gonzalez E, Greeves W. 2003. Marine invasive alien species: a threat to global biosecurity. Mar Policy. 27:313–323.

[cit0007] Bézy VS, Valverde RA, Plante CJ. 2014. Olive ridley sea turtle hatching success as a function of microbial abundance and the microenvironment of in situ nest sand at Ostional, Costa Rica. J Mar Biol. Vol. 2014. Article ID 351921. 10. 10.1371/journal.pone.0118579 PMC4340935 25714355

[cit0008] Cabanes FJ, Alonso JM, Castellá G, Alegre F, Domingo M, Pont S. 1997. Cutaneous hyalohyphomycosis caused by Fusarium solani in a loggerhead sea turtle (Caretta caretta L.). J Clin Microbiol. 35(12):3343–3345. 9399554 10.1128/jcm.35.12.3343-3345.1997 PMC230182

[cit0009] Candan ED. 2018. Molecular identification of fungal isolates and hatching success of green turtle (*Chelonia mydas*) nests. Arch Microbiol 200: 911-919. 29480404 10.1007/s00203-018-1496-0

[cit0010] Clukey KE, Lepczyk CA, Balazs GH, Work TM, Li QX, Bachman MJ, Lynch JM. 2018. Persistent organic pollutants in fat of three species of Pacific pelagic longlife caught sea turtles: accumulation in relation to ingested plastic marine debris. Sci Total Environ. 610-611:402–411. 28806556 10.1016/j.scitotenv.2017.07.242

[cit0011] Das MP, Kumar S. 2014. Microbial deterioration of low density polyethylene by *Aspergillus* and *Fusarium* sp. Int J ChemTech Res. 6:299–305.

[cit0012] Donelly K, Waltzek TB, Wellehan Jr. JFX, Sutton DA, Wiederhold NP, Stacy BA. 2015. Phaeohyphomycosis resulting in obstructive tracheitis in three green sea turtles *Chelonia mydas* stranded along the Florida coast. DisAquat Org 113: 257–262. 10.3354/dao02843 25850403

[cit0013] Duan C, Qin Z, Yang Z, Li W, Sun S, Zhu Z, Wang X. 2016. Identification of pathogenic *Fusarium* spp. causing maize ear rot and potential mycotoxin production in China. Toxins. 8:186–203. 10.3390/toxins8060186 PMC4926152 27338476

[cit0014] Duncan EM, Broderick AC, Fuller WJ, Galloway TS, Godfrey MH, Hamann M, Limpus CJ, Lindeque PK, Mayes AG, Omeyer LCM, et al. 2018. Microplastic ingestion ubiquitous in marine turtles. Glob Chang Biol. 2018:1–9. 10.1111/gcb.14519 PMC6849705 30513551

[cit0015] Elshafie A, Al-Bahry SN, AlKindi AY, Ba-Omar T, Mahmoud I. 2007. Mycoflora and aflatoxins in soil, eggshells, and failed eggs of chelonia mydas at Ras Al-Jinz, Oman. Chelonian Conserv Biol. 6(2):267–270.

[cit0016] Flint M. 2013. Free-ranging sea turtle health. Chapter 14. In: Flint M, Wyneken J, Lohmann KJ, Musick JA, editors. The biology of sea turtles. Northwest Florida: Chapman-Hall CRC; p. 379–398.

[cit0017] Glazebrook JS, Campbell RSF. 1990. A survey of the diseases of marine turtles in northern Australia. I. Farmed Turtles. Dis Aquat Org. 9:83–95.

[cit0018] Glazebrook JS, Campbell RSF, Thomas AT. 1993. Studies on an ulcerative stomatitis - obstructive rhinits - pneumonia disease complex in hatchling and juvenile sea turtles *Chelonia mydas* and *Caretta caretta*. Dis Aquat Organ. 16:133–147.

[cit0019] Groner ML, Maynard J, Breyta R, Carnegie RB, Dobson A, Friedman CS, Froelich B, Garren M, Gulland FMD, Heron SF, et al. 2016. Managing marine disease emergencies in an era of rapid change. R Soc Publishing. 371:20150364. 10.1098/rstb.2015.0364 PMC4760146 26880835

[cit0020] Gücül Ö, Biyik H, Sahiner A. 2010. Mycoflora identified from loggerhead turtle Caretta caretta) egg shells and nest sand at Fethiye beach, Turkey. Afr J Microbiol Res. 4(5):408–413.

[cit0021] Hamann M, Fuentes MMPB, Ban NC, Mocellin VJL. 2013. Climate change and marine turtles, chapter 13. In: Flint M, Wyneken J, Lohmann KJ, Musick JA. editors. The biology of sea turtles. Northwest Florida: Chapman-Hall CRC. p. 353–378

[cit0022] Harvell D, Altizer S, Cattadori IM, Harrington L, Weil E. 2009. Climate change and wildlife diseases: when does the host matter the most? Ecology. 90:912–920. 19449685 10.1890/08-0616.1

[cit0023] Hawkes LA, Broderick AC, Godfrey MH, Godley BJ. 2009. Climate change and marine turtles. Endangered Species Res. 7:137–154.

[cit0024] Huang Y, Busk P, Lange L. 2015. Cellulose and hemicellulose-degrading enzymes in *Fusarium commune* transcriptone and functional characterization of three identified xylanases. Enzyme Microb Technol. 73-74:9–19. 26002499 10.1016/j.enzmictec.2015.03.001

[cit0025] Jackson FD, Jin X, Schmitt JG. 2009. Fungi in a lower cretaceous turtle egg from China: evidence of ecological interactions. Palaios. 24:840–845.

[cit0026] Keene E, Soule T, Paladino F. 2014. Microbial isolation from olive ridley (*Lepidochelys olivacea*) and east pacific green (*Chelonia mydas agassizii*) sea turtle nests in pacific Costa Rica, and testing of cloacal fluid antimicrobial properties. Chelonian Conserv Biol. 13(1):1–7.

[cit0027] Lafferty KD. 2009. The ecology of climate change and infectious disease. Ecology. 90:888–900. 19449681 10.1890/08-0079.1

[cit0028] Lemmens M. 2012. *Fusarium* and Mykotoxine- Gefahr für Tier? Stapfia. 6:211–218.

[cit0029] Lowe RGT, McCorkelle O, Bleackley M, Collins C, Faou P, Mathivanan S, Anderson M. 2015. Extracellular peptidases of the cereal pathogen *Fusarium graminearum*. Front Plant Sci. 6:962. 26635820 10.3389/fpls.2015.00962 PMC4645717

[cit0030] Matiddi M, Hochsheid S, Camedda A, Baini M, Cocumelli C, Serena F, Tomassetti P,Travaglini A, Marra S, Campani T, et al. . 2017. Loggerhead sea turtles (Caretta caretta): A target species for monitoring litter ingestion by marine organisms in the mediterranean sea. Environ Pollut. 230:199–209. 28651091 10.1016/j.envpol.2017.06.054

[cit0031] Mo CL, Caballero M, Salas I. 1992. Microorganism infection of olive ridley eggs. In: Richardson JI, Richarson TH compilers, editors. Proceedings of the Twelfth Annual Workshop on Sea Turtle Biology and Conservation; February 25–29. Jeckyll Island, Georgia, NOAA Technical Memorandum NMFS-SEFSC-361: 274. p. 81–84.

[cit0032] Montero N, Tomillo PS, Saba VS, Dei Marcovaldi MAG, López-Mendilaharsu M, Santos AS, Fuentes MMPB. 2019. Effects of local climate on loggerhead atchling production in Brazil: implications from climate change. Sci Rep. 9:8861. 31222177 10.1038/s41598-019-45366-x PMC6586835

[cit0033] Mrosovsky N, Bass A, Corliss LA, Richardson JI, Richardson TH. 1992. Pivotal and beach temperatures for hawksbill turtles nesting in Antigua. Can J Zool. 70:1920–1925.

[cit0034] Neves MSC, de Melo Moura CC, de Oliveira LG. 2015. Mycobiota from the eggs, nests and stillbirths of eretmochelys imbircata linneus 1766 (Testudines: cheloniidae) in Pernambuco State, Brazil. Afr J Microbiol Res. 9(17):1195–1199.

[cit0035] O’Donnell K, Sutton DA, Rinaldi MG, Gueidan C, Crous PW, Geiser DM. 2009. Novel multilocus sequence reveals high genetic diversity of human pathogenic members of the *Fusarium incarnatum-F. equiseti* and *F. chlamydosporum* species/complexes within the United States. J Clin Microbiol. 47(12):3851–3861. 19828752 10.1128/JCM.01616-09 PMC2786663

[cit0036] O’Donnell K, Ward TJ, Robert VARG, Crous PW, Geiser DM, Kang S. 2015. DNA sequence-based identification o6f *Fusarium*: current status and future directions. Phytoparasitica. 43:583–595.

[cit0037] Oberbeckmann S, Löder MGJ, Labrenz M. 2015. Marine plastic-associated biofilms - a review. Environ Chem. 13:551–562.

[cit0038] Olivieri F, Zanetti ME, Oliva CR, Voarrubias AA, Casalongué CA. 2002. Characterization of an extracellular serine protease of Fusarium eumartii and its action on pathogenesis related proteiins. Eur J Plant Pathol. 108:63–72.

[cit0039] Özdemir B, Türkozan O. 2006. Hatching success of original and hatchey nests of the green turtle, *Chelonia mydas*, in northern Cyprus. Turk J Zool. 30:377–381.

[cit0040] Orós J, Arencibia A, Fernández L, Jensen HE. 2004a. Intestinal candidiasis in a loggerhead sea turtles (*Caretta caretta*): an immunohistochemical study. Vet J. 167:202–207. 14975396 10.1016/S1090-0233(03)00111-4

[cit0041] Orós J, Delgado C, Fernández L, Jensen HE. 2004b. Pulmonary hyalophyphomycosis caused by *Fusarium* spp in a Kemp’s ridley sea turtle *(Lepidochelys kempi)*: an immunohistochemical study. N Z Vet J. 52:150–152. 15768112 10.1080/00480169.2004.36420

[cit0042] Phillott AD, Parmenter CJ. 2001. Influence of diminished respiratory surface area on survival of sea turtle embryos. J Exp Zool. 289:317–321. 11241402 10.1002/1097-010x(20010415/30)289:5<317::aid-jez5>3.0.co;2-0

[cit0043] Phillott AD, Parmenter CJ. 2006. The ultrastructure of sea turtle egshell does not contribute to interspecies variation in fungal invasion of the egg. Can J Zool. 84:1339–1344.

[cit0044] Phillott AD, Parmenter CJ. 2014. Fungal colonization of green sea turtle (*Chelonia mydas*) nests is unlikely to affect hatchling condition. Herpetological Conserv Biol. 9(2):297–301.

[cit0045] Phillott AD, Parmenter CJ, Limpus CJ. 2004. The occurrence of mycobiota in eastern australian sea turtle nests. Mems Queensl Mus. 49:701–703.

[cit0046] Phillott AD, Parmenter CJ, Limpus CJ, Harrower KM. 2002. Mycobiota as acute and chronic cloacal contaminants of female sea turtles. Aust J Zool. 50:687–695.

[cit0047] Phillott AD, Parmenter CJ, McKillup SC. 2006. Calcium depletion of eggshell after fungal invasion of sea turtle eggs. Chelonian Conserv Biol. 5(1):146–149.

[cit0048] Ray AK, Guha MK, Sinha NK. 1982. Purification and characterization of an acidic trypsin/subtilisin inhibitor from tortoise egg white. Biochimica Et Biophysica Acta- General Subjects. 716:126–132. 10.1016/0304-4165(82)90260-4 7046804

[cit0049] Reynolds HT, Raudabaugh DB, Lilje O, Matthew C, Allender MC, Miller AM, Gleason FH. 2017. Chapter 27, Emerging mycoses and fungus-like diseases of vertebrate wildlife. In: Dighton J, White JF, editors. The fungal community: its organization and role in the ecosystem. 4th ed. Boca Raton (FL, USA): CRC Taylor and Francis; p. 286–403.

[cit0050] Sandoval-Denis M, Lombard L, Crous PW. 2019. Back to the roots: a reappraisal of neocosmospora. Persoonia. 43:90–185. 32214499 10.3767/persoonia.2019.43.04 PMC7085857

[cit0051] Sarmiento-Ramirez JM, Abella E, Martin MP, Telleria MT, López-Jurado LF, Marco A, Diéguez-Uribeondo J. 2010. *Fusarium solani* is responsible for mass mortalities in nests of loggerhead sea turtle, *Caretta careta* in Boavista, Cape Verde. FEMS Microbiol Lett. 312:192–200. 20875054 10.1111/j.1574-6968.2010.02116.x

[cit0052] Sarmiento-Ramirez JM, Abella-Pérez E, Phillott AD, Sim J, van West P, Martin MP, Marco A, Diéguez-Uribeondo J. 2014a. Global distribution of two fungal pathogens threatening endangered sea turtles. PLoS One. 9(1):e85853. 24465748 10.1371/journal.pone.0085853 PMC3897526

[cit0053] Sarmiento-Ramirez JM, Sim J, Van West P, Dieguez-Uribeondo J. 2017. Isolation of fungal pathogens from eggs of the endangered sea turtle species *Chelonia mydas* in Ascension Island. J Mar Biol Assoc UK. 97:661–667.

[cit0054] Sarmiento-Ramirez JM, van der Voort M, Raaijmakers JM, Diéguez-Uribeondo J. 2014b. Unraveling the microbiome of eggs of the endangered sea turtle *Eretmochelus imbracata* identifies bacteria with activity against the emerging pathogen *Fusarium falciforme*. PLoS One. 9(4):e 95205. 10.1371/journal.pone.0095206 PMC3990731 24743166

[cit0055] Short DPG, O’Donnell K, Thrane U, Nielsen KF, Zhang N, Juba JH, Geiser DM. 2013. Phylogenetic relationships among members of the *Fusarium solani* species complex in human infections and the descriptions of *F. keratoplasticum* sp nov. and *F. petroliphilum* stat. nov. Fungal Genet Biol. 53:58–70. 10.1016/j.fgb.2013.01.004 23396261

[cit0056] Sidique SNM, Azuddin NF, Joseph J. 2017. First report of *Fusarium* species at nesting sites of endangered sea turtles in Terengganu and Melaka, Malaysia. Malaysian Appl Biol. 46(3):195–205.

[cit0057] Smyth CW, Sarmineto-Ramírez JM, Short DPG, Diéguez-Uribeondo J, O’Donnell K, Geiser DM. 2019. Unraveling the ecology and epidemiology of an emerging fungal disease, sea turtle egg fusariosis (STEF). PLoS Pathog. 15(5):e1007682. 31095638 10.1371/journal.ppat.1007682 PMC6521983

[cit0058] Spatafora JW, Sung G-H, Johnson D, Hesse C, O’Rourke J, Serdani M, Spotts R, Lutzoni F, Hofstetter V, Maidlikowska J, et al. 2006. A five-gene phylogeny of pezizomycotina. Mycologia. 98:1018–1028. 17486977 10.3852/mycologia.98.6.1018

[cit0059] Wiles M, Rand TG. 1987. Integumental ulcerative disease in a loggerhead turtle *Caretta caretta* at the bermuda aquarium: microbiology and histopathology. Dis Aquat Organ. 3:85–90.

[cit0060] Zhang N, Castlebury LA, Miller AN, Huhndorf SM, Schoch C, Seifert KA, Rossman AY, Rogers JD, Kohlmeyer J, Volkmann-Kohlmeyer B, et al. 2006a. An overview of the systematics of the sordariomycetes based on a four-gene phylogeny. Mycologia. 98:1076–1087. 17486982 10.3852/mycologia.98.6.1076

[cit0061] Zhang N, O’Donnell L, Sutton DA, Nalim FA, Summerbell RC, Padhye AA, Geiser DM. 2006b. Members of the *Fusarium solani* species complex that cause infections in both humans and plants are common in the environment. J Clin Microbiol. 44(6):2186–2190. 16757619 10.1128/JCM.00120-06 PMC1489407

[cit0062] Zhang S, Ahearn DG, Nobel-Wang JA, Stulting RD, Schwam BL, Simmmons RB, Pierce GE, Crow A Jr. 2006c. Growth and survival of *Fusarium solani-F. oxysporum* complex on stressed multipurpose contact lens care solution films on plastic surfaces in Situ and In Vitro. J Cornea External Dis. 25:1210–1216. 10.1097/ICO.0b013e31802dd3a4 17172900

